# Delayed Presentation of Vaginal Cuff Dehiscence after Robotic Hysterectomy for Gynecologic Cancer: A Case Series and Review of the Literature

**DOI:** 10.1155/2016/5296536

**Published:** 2016-03-27

**Authors:** Pamela A. Escobar, Gregory M. Gressel, Gary L. Goldberg, Dennis Yi-Shin Kuo

**Affiliations:** Montefiore Medical Center and Albert Einstein College of Medicine, Department of Obstetrics & Gynecology and Women's Health, 1825 Eastchester Road, Room 722, Bronx, NY 10463, USA

## Abstract

*Background*. Vaginal cuff dehiscence after hysterectomy has varying incidence according to surgical approach, with highest rates associated with laparoscopic surgery. Comparative data on timing of diagnosis describe a wide range of clinical presentation from weeks to years after hysterectomy. Limited reports have focused specifically on delayed presentation of vaginal cuff dehiscence.* Cases*. All cases of vaginal cuff dehiscence at our institution between 2005 and 2015 were collectively reviewed and three cases were identified of women who presented with cuff dehiscence greater than 180 days from index surgery. Diagnosis occurred at 342 to 461 days after operation. One patient presented with abdominal pain, a second case presented with vaginal discharge, and the third case lacked clinical symptoms altogether. Prior to diagnosis, one case received chemotherapy and external beam radiation for Stage IB1 cervical cancer and another case received external beam radiation alone for Stage II endometrioid adenocarcinoma. All cuffs were repaired vaginally with interrupted, early absorbable suture.* Conclusion*. Robotic total laparoscopic hysterectomy may be associated with increased risk of vaginal cuff dehiscence. Further studies are needed to determine risk factors and patient characteristics associated with delayed presentation of vaginal cuff dehiscence in robotic total hysterectomy as well as all surgical approaches.

## 1. Introduction

Since its approval by the Food and Drug Administration in 2005, robotic surgery has increased in popularity and volume for both benign and malignant gynecological procedures. Well-described advantages include improved depth perception, enhanced ergonomics, autonomous camera and instrument control, and facilitation of complex laparoscopic maneuvers such as suturing [1]. Despite its increasing prevalence, the American College of Obstetricians and Gynecologists (ACOG) recommends total vaginal hysterectomy (TVH) as the primary surgical approach for benign disease whenever possible, as a large body of evidence demonstrates its advantages and lower complication rates [2]. Regarding gynecologic malignancies, ACOG highlights the paucity of prospective trials comparing robotic, laparoscopic, and open abdominal approaches that optimally define outcomes [3].

Vaginal cuff dehiscence (VCD) is a rare but serious complication of total hysterectomy. A robust literature review by Cronin and colleagues of VCD among all types of hysterectomy revealed incidence rates ranging from 0.14 to 4.1%, with higher incidence rates among robotic and total laparoscopic hysterectomy (TLH) at 1–4.1% [4]. Two recent case series of patients undergoing only robotic total hysterectomy described rates of 0.4% and 1.6% [5, 6]. The majority of large publications on VCD have included retrospective cohort studies and case series that include all types of hysterectomy rather than examining robotic-assisted surgery alone [7–9]. Data on timing of diagnosis of vaginal cuff dehiscence for all approaches is variable, with reports as early as 3 days and as late as 30 years after surgery. A mean time to cuff dehiscence among all approaches ranged from 6.1 weeks to 1.6 years [10, 11]. In comparison, a 2013 prospective cohort series on robotic hysterectomies for gynecologic cancers over a five-year period had a mean time of presentation of 154 days, with a range of 58 to 300 days (Drudi et al.). We describe three cases of vaginal cuff dehiscence after robotic radical and total hysterectomy presenting greater than 180 days from the index hysterectomy (Table 1).

## 2. Case 1

A 43-year-old woman with a diagnosis of Stage IB1, Grade 2 squamous cervical cancer underwent a robotic-assisted radical hysterectomy and pelvic lymph node dissection (LND). The patient also wanted to undergo a bilateral salpingooophorectomy (BSO) secondary to personal concerns of future malignancy in these organs. Her BMI was 26 kg/m^2^, she had no history of diabetes mellitus, and she was an active smoker. Total operative time was 5 hours and 48 minutes without complications and vaginal closure was accomplished with polyglactin 910 in a continuous end-to-end suture. Blood loss measured 100 mL. Her hospital course was unremarkable and she did not require adjuvant chemotherapy or radiation treatment. On postoperative day 461, the patient presented to the emergency department with complaints of vaginal pain and sensation of a vaginal bulge after intercourse. On examination, omental tissue was noted extruding from a 2.5 cm midline cuff defect. The eviscerated omentum appeared without vascular compromise. The cuff was repaired vaginally with interrupted polyglactin 910 suture. A biopsy was taken to rule out local recurrence and returned consistent with squamous mucosa with focal epithelial erosion and acute and chronic inflammation involving stromal tissue.

## 3. Case 2

A 51-year-old woman with Stage IB1, Grade 3 squamous cervical cancer underwent an uncomplicated robotic radical hysterectomy, BSO, and pelvic LND lasting 6 hours and 4 minutes. Her BMI was 18 kg/m^2^, she had no toxic habits, and she did not have diabetes mellitus. Total blood loss was 250 mL and her vaginal cuff was closed with barbed suture in a bidirectional running fashion to the midline. Postoperative adjuvant treatment included 5 cycles of cisplatin, external beam radiation, and brachytherapy. She presented on postoperative day 342 for routine follow-up, and speculum examination incidentally revealed a 4 cm cuff defect with small bowel exposure. The cuff was repaired vaginally with interrupted polyglactin 910 suture. Pathology revealed fibroadipose tissue with focal chronic inflammation and foreign body giant cells.

## 4. Case 3 

A 70-year-old woman with Stage II, Grade 1 endometrioid adenocarcinoma underwent an uncomplicated robotic-assisted TLH, BSO, pelvic and para-aortic LND, and cystoscopy lasting 3 hours and 53 minutes. Her BMI was 28 kg/m^2^, she had no toxic habits, and she did not have diabetes mellitus. Total blood loss was 100 mL and her vaginal cuff was closed with polyglactin 910 in a bidirectional running fashion to the midline. Postoperative treatment included external beam radiation and brachytherapy. Although the patient was not sexually active, she did report use of vaginal dilators for prevention of vaginal stenosis. She presented as an outpatient on postoperative day 343 complaining of vaginal discharge for one month. On exam, a 1.5 cm cuff defect was visualized with straw-colored fluid extravasation and no evisceration. The cuff was repaired vaginally with interrupted polyglactin 910 suture without complications. No specimen was sent to pathology.

All hysterectomies were accomplished using the Intuitive Surgical® da Vinci Surgical System by experienced gynecologic oncologists at a single institution. Standard preoperative antibiotics were administered and postoperative healing, excluding VCD, was uncomplicated without evidence of infection. Circumferential colpotomies were made using monopolar curved scissors on coagulation mode following transection of uterine vessels. Specimens were evacuated through the colpotomy and vaginal cuff edges were reapproximated as described above. Knots were tied robotically.

## 5. Discussion

A total of 688 laparoscopic hysterectomies were performed by eight gynecologic oncologists for both benign and malignant disease at our institution between 2005 and 2015: 544 patients underwent robotic TLH and 144 patients underwent TLH. While the majority of the cases had a preoperative diagnosis of dysplasia or malignancy, indications for benign surgery included adnexal masses, endometriosis, and leiomyomatous uteri. During this period, three patients had a VCD less than 180 days following robotic TLH and three patients had VCD greater than 180 days following robotic TLH. In the laparoscopic group, two patients experienced VCD prior to 180 days and none experienced VCD greater than 180 days. The three cases described above are unique in that they describe vaginal cuff dehiscence greater than six months after initial hysterectomy. Delayed cuff dehiscence has rarely been reported in the relevant literature for robotic-assisted TLH. Prior to these cases, Drudi et al. presented seven cases of VCD in robotic hysterectomy for gynecological cancers, describing one case that presented at 300 days after operation following a robotic TLH, BSO, and pelvic and para-aortic LND for endometrial cancer. While no definitions of early versus late presentation of VCD have been established, mean time to presentation for robotic hysterectomy cases has been outlined at as high as 154 days. Because one would expect dissolution of absorbable suture and complete wound reapproximation by this time, we suggest that patients with delayed VCD may have other predisposing risk factors.

Several risk factors have been outlined for vaginal cuff dehiscence. Mode of hysterectomy has been frequently described as a contributing factor, with highest rates among laparoscopic procedures. Cohort studies have reported an incidence of 1.1–4.9% after TLH and 3% after robotic hysterectomy versus 0.29% and 0.12% after TVH and abdominal hysterectomy, respectively [12, 13]. Moreover, a 2012 cohort study concluded that vaginal closure of the cuff was associated with a threefold and ninefold decrease in risk of dehiscence compared with laparoscopic and robotic closure, respectively [14]. Lacking in the literature are randomized controlled trials comparing surgical approaches that are sufficiently powered to detect a clinical significance. Nonetheless, available reports in the form of case series and cohort studies suggest a higher likelihood of VCD among laparoscopic, compared to vaginal and abdominal, hysterectomies.

Another factor described as having an impact on risk of VCD is method of cuff closure. Electrocautery is used with greater frequency for colpotomy in laparoscopic procedures compared to vaginal and abdominal approaches, which has been postulated to lead to a decrease in vascularization essential for wound healing. Specific to electrocautery, the use of monopolar current on cutting mode has been hypothesized to contribute less thermal spread compared to coagulation mode. Several studies have outlined certain surgical techniques thought to decrease the risk of VCD after laparoscopic surgery, such as use of monopolar current on cutting mode for colpotomy, hemostasis via sutures versus electrocoagulation, and use of 2-layer cuff closure with polydioxanone suture [15–19]. A retrospective analysis of 134 patients undergoing robotic hysterectomy by a single surgeon examined vaginal cuff closure with 2-0 monofilament absorbable suture compared to 2-0 absorbable unidirectional barbed suture. The main difference between these sutures is the lack of knot-tying required with use of barbed suture as, theoretically, tension is controlled across tissue when the barbs are locked into place. Results demonstrated no cases of VCD or vaginal cuff cellulitis with comparable rates of spotting and bleeding [20]. At our institution, we employ monopolar curved scissors in coagulation mode for colpotomy with efforts to provide voltage over a minimum quantity of surface area to avoid thermal spread and maintain hemostasis. For vaginal closure, use of interrupted sutures rather than a single continuous suture is practiced and closures involving barbed and braided sutures were demonstrated in these cases.

Other risk factors associated with VCD include poor wound healing, increased intra-abdominal pressure, intraoperative or postoperative infection, and hematoma formation [21, 22]. Etiologies of poor wound healing include malignancy, tobacco use, chronic steroid use, malnutrition, or history of radiation [23]. However, studies evaluating these factors are limited and conflicting. One study demonstrated that no difference has been shown in age, tobacco use, or diabetes mellitus in women with and without VCD, although the low incidence of VCD (1.7%) was most likely contributory (Nick et al.). Unexpectedly, obesity and age were associated with a decreased risk of VCD following robotic and laparoscopic hysterectomies but not vaginal or abdominal routes. Obese women (BMI ≥ 30 kg/m^2^) were 86% less likely than women of normal weight (BMI < 25 kg/m^2^) to experience a VCD following laparoscopic surgery [24]. The BMIs for our three patients were within normal limits.

Adjuvant radiation therapy was given to 2 of 3 of these patients. Nevertheless, the two patients presented long after their adjuvant treatment. Only one patient had a precipitating event, intercourse, while another patient may have potentially perforated her cuff with use of vaginal dilators. Drudi et al. present a series in which patients who had received postoperative adjuvant treatment (chemotherapy and/or brachytherapy) had a higher rate of vaginal dehiscence versus those who did not have any adjuvant treatment, 3% and 0.4%, respectively. In our study, the case that did not receive postoperative adjuvant treatment occurred immediately postcoitally. None of our patients were diabetic and only one was an active smoker. A histologic study examining tissue characteristics in women with VCD after robotic hysterectomy and those without VCD found significantly higher levels of acute and inflammatory cells, suggesting a prolonged inflammatory phase that can impact reparative healing in patients with dehiscence [25]. Pathology for the initial two VCD cases in this report showed evidence of chronic inflammation. Despite identification of these risk factors, the causes for these three VCD cases are uncertain and likely multiple factors are involved. Patient characteristics and postoperative courses in our cases, however, did not differ from previous reports described in the oncologic population following robotic hysterectomy.

Diagnosis of VCD requires prompt surgical management. In up to 70% of cases, evisceration of bowel or intra-abdominal contents occurs with the distal ileum being the most frequent eviscerating organ [7, 26]. Presenting symptoms include pelvic or abdominal pain and vaginal bleeding or watery discharge, but VCD can also present as an incidental finding at routine follow-up. Sexual intercourse and excessive valsalva have been described as precipitating factors [27, 28]. In our case series, only one patient had an identifiable precipitating event: coitus. Two patients presented with symptoms while one patient was diagnosed incidentally on exam. No evidence-based research is available to guide recommendations for avoidance of precipitating events, specifically coitus. In our practice, patients are asked to avoid sexual intercourse for at least six to eight weeks until vaginal cuff evaluation as an outpatient is determined to be satisfactory. Given that the available information on VCD is mostly derived from case reports and case series, the impact of risk factors by type of procedure (robotic TLH compared to TLH) is difficult to differentiate. Thus, preoperative counseling regarding risks of surgery as well as risk factors for VCD is similar for both types of procedures. Our patient with coitus as a precipitating event was able to resume sexual intercourse following VCD repair without recurrence. The remaining patients had uncomplicated repairs without recurrence of symptoms.

Methods of surgical repair include abdominal, vaginal, or laparoscopic approaches, but current data has not determined superiority of closure method. Generally, management of VCD involves emergent vaginal repair unless there is difficulty with reduction of eviscerated abdominal contents or evidence of bowel compromise [29]. Successful outcomes, however, have been described using vaginal or combined vaginal and laparoscopic approaches in selected cases of vaginal cuff evisceration if there is no evidence of peritonitis or ischemic injury to the prolapsed bowel segment [30]. Factors impacting method of repair include hemodynamic stability, clinical evidence of peritonitis or bowel injury, and individual surgical skill.

With increased prevalence of robotic surgery, there is a growing body of literature evaluating treatment and patient factors in robotic TLH for both benign and malignant disease. There still remains a paucity of data regarding VCD remote from surgery, warranting further analysis of patient-related risk factors as well as surgical and nonsurgical treatments. Our small retrospective number is limited to description of surgical techniques and patient characteristics in an oncologic population. However, these findings highlight the vigilance warranted for VCD more than 11 months after robotic surgery. Patients with malignancy should be counseled on the risk of VCD with deep penetration of the vault, especially those receiving adjuvant chemotherapy and/or radiation.

## Figures and Tables

**Table 1:** Characteristic of patients with vaginal vault dehiscence.

Pt	Age	BMI (kg/m^2^)	Diagnosis	POD	Presenting symptoms	Defect	Initial vaginal cuff closure	Method of reclosure	Risk factors
1	43	26	Squamous cell cervical cancer	461	Vaginal pain, vaginal bulge	2.5 cm	Polyglactin 910, continuous end-to-end suture	Polyglactin 910, interrupted suture	Coitus Active smoker

2	51	18	Squamous cell cervical cancer	342	None	4 cm	Barbed suture, bidirectional running fashion	Polyglactin 910, interrupted suture	EBRT

3	70	28	Endometrioid adenocarcinoma	343	Vaginal discharge	2.5 cm	Polyglactin 910, bidirectional running fashion	Polyglactin 910, interrupted suture	EBRT Brachytherapy Vaginal dilator use

Pt, patient; BMI, Body Mass Index; POD, postoperative day; EBRT, External Beam Radiation Therapy.
